# Cutaneous Erythema at Scar Site of Modified Radical Mastectomy: An Unexpected Manifestation of Recurrent Carcinoma

**DOI:** 10.1155/2017/6879626

**Published:** 2017-08-10

**Authors:** Awrad Mohammed-Reda Nasralla, Mohammed Abdulirazzaq Al-Duhileb, Ali Jamal-Aldein Arini, Samir Sami Amr

**Affiliations:** ^1^Department of Surgery, King Fahad Specialist Hospital, Dammam, Saudi Arabia; ^2^Department of Pathology and Laboratory Medicine, King Fahad Specialist Hospital, Dammam, Saudi Arabia

## Abstract

A 44-year-old woman presented with marked erythema over right mastectomy scar, while on Herceptin therapy. She had neoadjuvant chemotherapy, modified radical mastectomy, and radiotherapy less than one year earlier for the treatment of invasive ductal carcinoma. On physical examination, no palpable masses were detected in the erythematous skin. A biopsy revealed permeation of the skin lymphatics by emboli of metastatic ductal carcinoma, similar to what is seen in inflammatory carcinoma. The involved skin was excised, followed by immediate reconstruction with transverse rectus abdominis muscle (TRAM) flap. On follow-up, the wound was healing well, with no signs of inflammation.

## 1. Introduction

Five to ten percent of patients with operable breast cancer develop a chest wall recurrence within 10 years following mastectomy [1, 2]. Local skin recurrence is uncommon and is believed to manifest as hard nodule, but it is rare to present as rash or erythema [3, 4]. We present herein a patient with skin rash within less than a year following neoadjuvant chemotherapy, mastectomy, and adjuvant radiotherapy, while she was still receiving adjuvant biological therapy.

## 2. Case Report

A 44-year-old woman had invasive ductal carcinoma of the right breast. On initial presentation, she had a palpable 7 × 5 cm well-circumscribed hard mass at upper outer quadrant of the right breast with no skin changes nor tethering or attachment to the underlying muscle. There were no other ipsilateral or contralateral palpable masses. The right axilla had a palpable 1 cm lymph node. Core biopsy of the breast mass revealed invasive ductal carcinoma, ER/PR negative, and HER-2 positive. The US-guided FNAC of the right axillary lymph node confirmed the presence metastatic breast cancer. She received neoadjuvant chemotherapy and biological therapy; then, she underwent modified radical mastectomy followed by radiotherapy. Pathological examination of the mastectomy specimen revealed a 3 cm tumor nodule that showed on histological examination invasive ductal carcinoma, with ductal carcinoma in situ, comedo type, grade 3, ER/PR negative, and Her2-neu positive. The tumor had clear surgical margins, and 9 axillary lymph nodes were all negative for malignancy.

**Figure 1:**
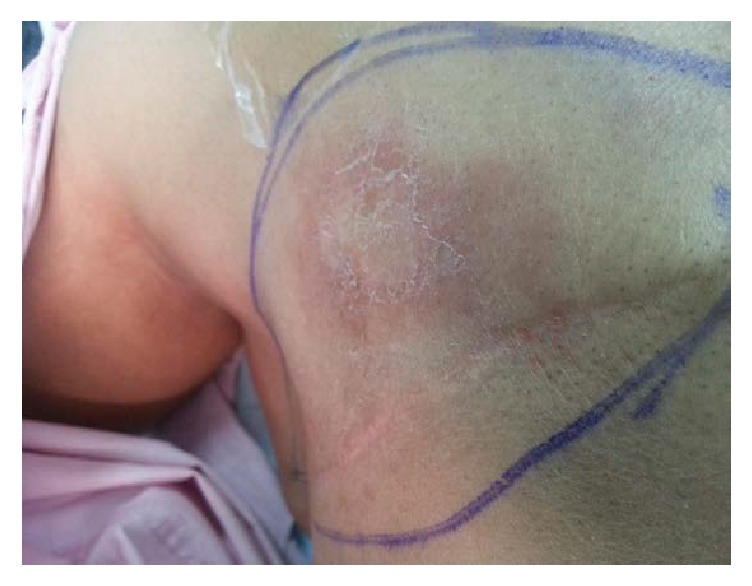
Preoperative picture of the skin lesion on the right anterior chest over the mastectomy scar.

Six months following the surgery, she presented with redness over the mastectomy scar that increased in size rapidly within 2 months. On physical examination, the skin lesion was a localized, well-defined, indurated erythematous not warm plaque measuring 5 × 4 cm overlying the right anterior chest wall over the mastectomy scar (Figure 1).

Initially, it was thought that the skin change was related to the radiotherapy, but the skin lesion increased in size and the patient was referred to Dermatology Clinic for consultation. She was given topical corticosteroid and antifungal agents, without clinical improvement. A punch biopsy was performed, and it revealed recurrent breast invasive ductal carcinoma, manifesting as dermal lymphatic tumor emboli.

**Figure 2:**
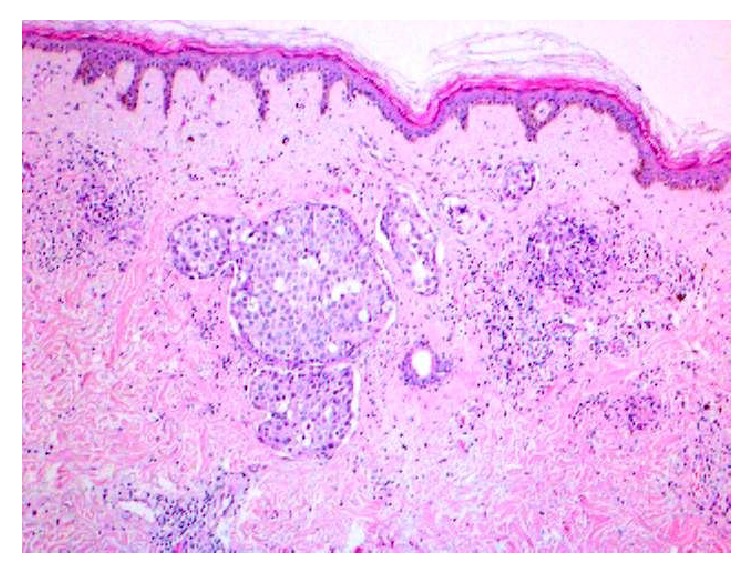
Histopathology of the skin lesion featuring intact epidermis and several dilated lymphatics or capillaries in the dermis filled with tumor emboli (H&E ×40).

**Figure 3:**
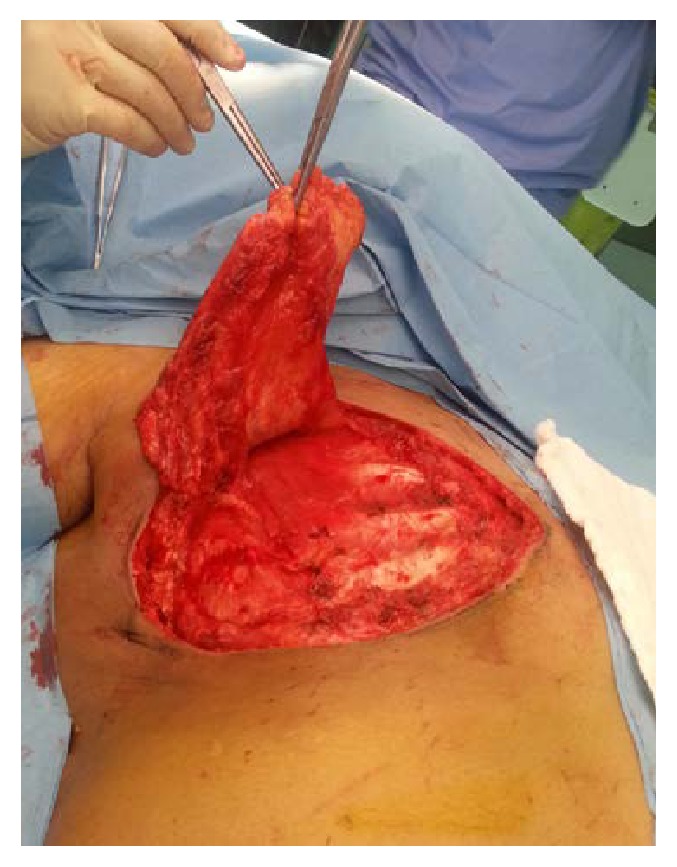
Intraoperative picture of the resected tumor.

Accordingly, the involved skin was excised with a wide local margin, and the pectoralis major muscle was removed en block, followed by immediate reconstruction with TRAM flap (Figure 2). The aim of the flap is to close the wound primarily and to have a better cosmetic outcome. Histopathological study of the resected tumor showed extensive tumor emboli within the lymphatics of the dermis of the involved skin (Figure 3). Surgical margins of the skin and the pectoralis major muscle were free from the tumor.

## 3. Discussion

Breast cancer metastasis to the skin is uncommon, with 0.6% to 9% of the patients developing such metastases within 2 to 3 years. It usually presents as a firm nodule at the site of the primary cancer that can be associated with ulceration, bleeding, and pain [3–5].

Radiotherapy following mastectomy decreases the rate of the chest wall recurrence by more than 50% [1, 6]. However, differential diagnosis that should be considered following mastectomy and radiotherapy is atypical vascular lesions that occur in the skin within the radiation field. It is a rare condition that develops months to years following radiotherapy in the form of papules or erythematous patches or plaques. It has a benign course; however, it has been reported that such lesion could progress to angiosarcoma [7–9].

However, our patient initially had noninflammatory stage IIIA right breast cancer (T3N1M0) and received the standard treatment. Then shortly, she presented to our clinic complaining of redness over the mastectomy scar, with no visible mass, or ulcer, nor bleeding. The skin lesion developed within short period following the radiotherapy. It was a challenge to diagnose such a case given the following factors: the noninflammatory breast cancer, the good response to chemotherapy, and the rapid recurrence despite the given chemotherapy and radiotherapy treatment.

Management of local recurrence is challenging due to the lack of randomized control trials [1]. In this case, the recurrent tumor was excised with wide local margin, and the pectoralis major muscle was removed en block, followed by immediate reconstruction with TRAM flap. Local recurrence after mastectomy rarely presents as isolated skin lesion in a form of erythematous rash with no palpable mass. Here, we are emphasizing the role of history and physical examination to detect local recurrence following mastectomy, since the imaging could be less clear after mastectomy.

## 4. Conclusion

Isolated skin recurrence in the form of erythema does occur in breast cancer patients following mastectomy. History and physical examination are important in the diagnosis of recurrence of breast cancer. In this case, images could miss this kind of skin recurrence, and we recommend getting a biopsy to establish a diagnosis.

## References

[1] Hirsch A., Sabel M. S., Hayes D. F. (2015). *Management of Locoregional Recurrence of Breast Cancer after Mastectomy*.

[2] Buchanan C. L., Dorn P. L., Fey J. (2006). Locoregional recurrence after mastectomy: incidence and outcomes. *Journal of the American College of Surgeons*.

[3] Chernoff K. A., Marghoob A. A., Lacouture M. E., Deng L., Busam K. J., Myskowski P. L. (2014). Dermoscopic findings in cutaneous metastases. *JAMA Dermatology*.

[4] Sariya D., Ruth K., Adams-McDonnell R. (2007). Clinicopathologic correlation of cutaneous metastases: Experience from a cancer center. *Archives of Dermatology*.

[5] Cabula C., Campana L. G., Grilz G., Galuppo S., Bussone R., De Meo L. (2015). Electrochemotherapy in the treatment of cutaneous metastases from breast cancer: a multicenter cohort analysis. *Annals of Surgical Oncology*.

[6] Azrif M., Saladina J. J., Nani M. L., Shahrunniza A. S., Norlia A., Rohaizak M. (2011). Isolated late chest wall recurrence after mastectomy for breast cancer. *Medical Journal of Malaysia*.

[7] Jayalakshmy P. S., Sivaram A. P., Augustine J., Bindu P. (2012). Postmastectomy-postirradiation atypical vascular lesion of the skin: report of 2 cases. *Case Reports in Pathology*.

[8] Gengler C., Coindre J.-M., Leroux A. (2007). Vascular proliferations of the skin after radiation therapy for breast cancer: Clinicopathologic analysis of a series in favor of a benign process - A study from the French Sarcoma Group. *Cancer*.

[9] Patton K. T., Deyrup A. T., Weiss S. W. (2008). Atypical vascular lesions after surgery and radiation of the breast: a clinicopathologic study of 32 cases analyzing histologic heterogeneity and association with angiosarcoma. *American Journal of Surgical Pathology*.

